# SARS-CoV-2 indoor air transmission is a threat that can be addressed with science

**DOI:** 10.1073/pnas.2116155118

**Published:** 2021-11-02

**Authors:** Jonathan M. Samet, Thomas A. Burke, Seema S. Lakdawala, John J. Lowe, Linsey C. Marr, Kimberly A. Prather, Marilee Shelton-Davenport, John Volckens

**Affiliations:** ^a^Office of the Dean, Colorado School of Public Health, Aurora, CO 80045;; ^b^Department of Health Policy and Management, Johns Hopkins Bloomberg School of Public Health, Johns Hopkins University, Baltimore, MD 21205;; ^c^Department of Microbiology & Molecular Genetics, University of Pittsburgh, Pittsburgh, PA 15219;; ^d^Department of Environmental, Agricultural & Occupational Health, University of Nebraska Medical Center, Omaha, NE 68198;; ^e^Department of Civil and Environmental Engineering, Virginia Polytechnic Institute and State University, Blacksburg, VA 24061;; ^f^Department of Chemistry and Biochemistry, Scripps Institution of Oceanography, University of California San Diego, La Jolla, CA 92093;; ^g^National Academies of Sciences, Engineering, and Medicine, Washington, DC 20001;; ^h^Department of Mechanical Engineering, Colorado State University, Fort Collins, CO 80523

The Environmental Health Matters Initiative (EHMI) of the National Academies of Science, Engineering, and Medicine (NASEM) was established by the three presidents as a mechanism for a transformational cross-institutional approach to enable challenges to be informed—rapidly if needed—by insights from a broad range of applicable scientific disciplines and sectors spanning academia, government, foundations, businesses, and nongovernmental organizations. The EHMI reaches across the three National Academies to provide a venue for bringing transdisciplinary and cross-sector thinking to environmental health issues that are urgent, on the horizon, or recalcitrant in nature. This paper describes how EHMI approached the critical and extremely vexing problem of the airborne transmission of severe acute respiratory syndrome coronavirus 2 (SARS-CoV-2) as the pandemic reached the six-month mark in the United States.

From its emergence in 2019 and identification in early 2020 the SARS-CoV-2 virus spread quickly and globally, causing multiple waves of infection and the disease COVID-19. The spread of the virus has been facilitated by its transmission dynamics, with several days of virus shedding before symptoms emerge. With the pandemic surging in early 2020, researchers throughout the world addressed how it was transmitted, turning to established concepts: by contact with contaminated surfaces, so-called fomite transmission, by larger particles generally termed “droplets,” and by smaller particles referred to as “aerosols.” At the pandemic’s start, there was great uncertainty about the transmission of SARS-Cov-2 and, consequently, the pace of research on airborne transmission was greatly accelerated by the COVID-19 pandemic.

In the context of a global pandemic causing millions of deaths and halting economies, implementation of effective transmission control measures was urgent. Critical to transmission control was the relative impact of the three modes of transmission, each leading to different control strategies: fomites by cleaning of surfaces and hand hygiene; droplets by physical distancing and facial coverings; and aerosols by facial coverings, physical distancing, ventilation of indoor spaces, and air cleaning via filtration. Given implications of limited resources and the costs of implementing these different strategies, it was crucial to properly recognize these modes, especially aerosols, whose role had been downplayed due to widespread misunderstanding about airborne transmission. To overcome this gap, there was a surge of research on airborne transmission, leading to evidence that began to point toward a key role for aerosols. However, controversy on airborne transmission continued into the summer of 2020, even as more research findings were reported, but authoritative reviews had not been conducted. At that time, neither the World Health Organization (WHO) nor the Centers for Disease Control and Prevention (CDC) had supported airborne transmission by aerosols, giving more emphasis to large droplets.

Consequently, the EHMI convened a workshop in August 2020 on a rapid timeline to address this cross-disciplinary issue at the intersection of environmental considerations, public health, and clinical care. Reflecting the urgency of the COVID-19 pandemic and of achieving understanding of the transmission modes, the workshop was conceived, framed, and carried out virtually in ∼8 wk with the input of National Academies’ committee volunteers and staff, aligning with the broader suite of NASEM work on SARS-CoV-2 challenges. The workshop was organized to address questions that needed to be answered for decision-making.

Here, we provide a high-level synthesis of answers to the key questions considered in the workshop Airborne Transmission of SARS-CoV-2 (https://www.nationalacademies.org/event/08-26-2020/airborne-transmission-of-sars-cov-2-a-virtual-workshop). Details on the evidence considered, the complexities of terminology, and the judgments provided by participating experts are captured in the Proceedings in Brief (1).

## Science Clarified by the Workshop

**Fig. 1.**
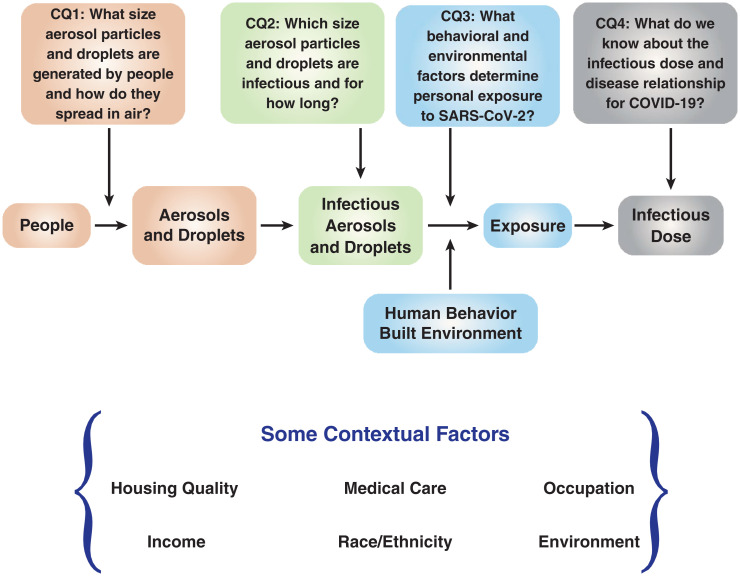
Framework for organizing the workshop. This framework is consistent with the National Academies’ series of reports on *Research Priorities for Airborne Particulate Matter* (16). Reprinted with permission from ref. 17.

As the workshop was planned there were many unresolved questions related to airborne transmission of SARS-CoV-2. Some were related to pathogenesis: How many virions were needed to cause infection?; some to the mechanisms of airborne transmission: How were infectious aerosol particles generated and which aerosol particle sizes contained viable virus?; and some to control measures: What level of ventilation and filtration is needed in buildings, do face coverings work, and does air filtration reduce transmission? As a first step in organizing the workshop to address the most critical questions a framework was developed (Fig. 1), based on the long-standing public health paradigm of moving from source of the agent of concern to exposure and dose and on to injury and disease. The framework acknowledged that there are cross-cutting societal issues (contextual factors in the diagram) that needed to be reflected in workshop discussions, particularly the inequities of the pandemic.

In this schema (Fig. 1), infected people are the source, generating aerosols and droplets that contaminate the air. Airborne exposure occurs when people inhale virus-containing aerosols and droplets traveling in air. The dose constitutes the inhaled virions that are deposited in the respiratory tract at points beginning with the upper respiratory tract—nose, oropharynx, and larynx—and extending down into the lower respiratory tract—trachea, bronchi, bronchioles, and alveoli. The planning committee identified four critical questions (Fig. 1) in this source-to-dose paradigm and used these questions to frame the workshop.

The workshop planning committee recognized the already substantial body of evidence on airborne transport, deposition, and exposure to aerosols, including respiratory viruses, that is relevant to the transmission of SARS-CoV-2. Much is known concerning the behavior of airborne particulate matter (PM) or aerosol particles suspended in the air, respiratory tract defenses, and the probability of PM deposition at sites within the respiratory tract based on size of the inhaled aerosol particles. Larger particles stay suspended in the air for many seconds to minutes and are deposited in the upper airway. Smaller particles can remain airborne for hours to days and travel long distances. These smaller particles can reach the lower respiratory tract upon inhalation. One outcome of the workshop planning and other discussions was the formulation of size-related definitions of aerosols and droplets in the context of transmission of infection and inhalation of aerosols. These definitions were reported in a separate publication (2).

Much was already known about the role of building ventilation and air cleaning in providing healthy indoor air (at least since the 1918 pandemic). The American Society for Heating, Refrigerating, and Air-Conditioning Engineers (ASHRAE) provides standards and guidelines covering air exchange needs for various types of spaces, including residences, commercial properties, and health care facilities. ASHRAE covers air cleaning as well. Frameworks for using personal protective equipment (PPE) for inhaled agents were in place for use in specific occupational settings, including health-care settings. However, both the science and practice of face coverings by the general public had not been adequately addressed by any organization.

While the foundation of evidence relevant to the four critical questions (Fig. 1) was already substantial as the workshop was planned, airborne transmission of SARS-CoV-2 had not been systematically examined in the context of the existing evidence. Some areas were particularly controversial, including the relative extent of transmission by droplets and aerosols, the role of building ventilation, and the effectiveness of masks. These controversies played out in real time on social media and in the tsunami of COVID-19 preprints during the pandemic.

For each critical question, presentations and panel discussions identified the key, relevant research. The moderators then synthesized the evidence presented for each critical question, summarizing “what we know” and providing the complementary discussion of “what is needed.” The workshop concluded with a multisectoral panel that highlighted the broad policy implications of findings on the critical questions.

### Critical Question 1: What Size Aerosol Particles and Droplets Are Generated by People and How Do They Spread in Air?

In seminal papers from the 1930s (3) Wells proposed the concept of droplets and droplet nuclei that has dominated thinking about airborne infection over the ensuing decades. Wells commented that larger droplets, governed by Stokes’ law, would fall quickly to the ground, while smaller droplet nuclei, formed from droplets that evaporated and left behind solid residue, would stay airborne for longer periods of time. Since then, studies in the field of aerosol science have shown that exhalation and various exhalatory activities, e.g., singing, variably generate thousands of small particles (of multiple size modes) that can travel well beyond 6 feet and accumulate in poorly ventilated indoor spaces.

The general discussion of airborne transmission of SARS-CoV-2 had been hindered by the terminology used to describe infectious respiratory droplets and aerosols, with long-standing concepts and definitions that defied physics. Workshop planning committee members and others proposed a terminology that was introduced in the keynote by Linsey Marr: “Droplets” are larger than 100 μm in aerodynamic diameter, whereas “aerosols” are smaller than 100 μm (2). This definition departs from the size threshold of 5 μm used in the health-care community for decades and more usefully captures the airborne behavior and transmission mode of infection. Large droplets are “sprayed,” travel only short distances, fall quickly, and generally settle within 6 feet of the source person; exposure occurs by impaction of droplets on the body. Aerosols, generated in multiple ways in the respiratory tract, are emitted via exhalation and by simply speaking and can remain suspended for minutes to hours—particularly those in the smaller size range—and travel across and accumulate in indoor spaces

The synthesis for the first question pointed to the need to update and unify terminology to better reflect the process of airborne transmission as proposed by Prather et al. (2). Aerosols can contain infectious virus with emission rates that vary by activity, individual, and stage of disease. The confluence of activities and individuals producing large numbers of aerosols creates the opportunity for superspreading events, such as the choir practice in Skagit, Washington (4).

Research was called for to advance understanding of the generation of infectious particles in relation to the characteristics and activities of infectious people and of the relative importance of aerosols and droplets and the viral loads they contain as a function of size. To better understand the relative contributions of factors controlling transmission by droplets and aerosols, methods are needed for sampling airborne virus that preserve infectivity.

### Critical Question 2: Which Size Aerosol Particles and Droplets Are Infectious and for How Long?

Research shows that aerosols, including those smaller than 5 μm, contain SARS-CoV-2 RNA, although the presence of RNA alone does not establish that infectious virus is present. One study was cited as definitively showing that aerosols can contain viable virus (5). In this study, which involved air sampling from a hospital room shared by two COVID-19 patients, viable virus was collected from the room air and the particles collected caused cytopathic damage in a cell culture system. The aerosols can stay suspended for hours and be dispersed widely by air currents in indoor spaces, although the rate of loss of viability under natural circumstances is not established.

Information gaps are abundant, reflecting the many environments of interest and the diverse factors that may affect aerosol concentration and viability of the virus. The synthesis suggested both experimental and observational approaches to improve the evidence foundation related to particle size, virus viability, and infection.

### Critical Question 3: What Personal and Environmental Factors Determine Personal Exposure to SARS-CoV-2?

Personal exposure comes from being in places where the air and surfaces are contaminated with virus. For airborne transmission, exposure can occur by inhaling aerosols at short range in the respiratory plume of an infected individual and at longer range in air that becomes contaminated as aerosols in the plume disperse. This longer-range transmission of aerosols is of particular concern in closed, crowded, and poorly ventilated spaces, a scenario contributing to superspreading events.

This understanding of exposure and the role of aerosols points to modifiable determinants of the level of exposure. Behavior change to avoid spaces prone to accumulating aerosols is an obvious step that, when possible, can be taken at the individual level or motivated by policy measures. Use of facial coverings is another individual-level step that can be a matter of choice or taken because of policy measures, e.g., community mask orders. The synthesis for this question noted sufficient evidence to conclude that masks provide source control, i.e., reducing the release of droplets and aerosols, and also provide some protection for their wearers.

Airborne transmission of SARS-CoV-2 by aerosols implies that infection risks can be reduced by making changes to the built environment: increasing exchange of indoor with outdoor air, air filtration, and germicidal ultraviolet irradiation. Limiting the number of people within spaces and distancing them reduces the strength of the source—people—and the intensity of the exposure.

The accumulating data on indoor and enclosed environments could facilitate modeling to sort out the determinants of exposure for occupants and the consequences of interventions. Enhanced measurement technologies would lead to more informative modeling by providing data on personal behaviors and particle size distributions. Risk reduction from changes to building, transit, and other enclosed environments could be quantified with a risk model that reflected the sequence of events in Fig. 1.

### Critical Question 4: What Do We Know About the Infectious Dose and Disease Relationship for COVID-19?

Answering this question is key for quantifying infection and disease risk and modeling the impact of control measures as they influence the dose of virions reaching receptors in the respiratory tract. Beyond determining whether infection occurs, the dose of virions delivered to the respiratory tract might also influence the clinical manifestations and severity of the resulting disease. There has been limited research related to this question for coronaviruses and much of our understanding comes from studies on influenza viruses. With experimental influenza virus infections in the ferret model the strain of virus is a determinant of the release and size distribution of exhaled virus-containing droplets and aerosols (6–9). We know that the size of virus-laden aerosols will contribute to the deposition of the virus within the respiratory tract and influence the anatomical site of infection initiation, which can impact both severity and success of the resultant infection. In addition, the infectious dose for influenza viruses can vary by viral subtype and route of infection.

Observational data for SARS-CoV-2 indicate a wide range of host factors that may influence infectious dose and disease risk and outcomes. Evidence describing the dose–response relationship and infectious dose was found to be largely lacking, although useful animal models have now been developed demonstrating that aerosols transmit infection in some species. Animal model studies on SARS-CoV have revealed that the infectious dose can vary based on the age of the animal, and epidemiological data have demonstrated a sex-based difference in COVID-19 severity. These data suggest that genetic factors and biological response to infection will influence the response to the dose received and the severity of SARS-CoV-2 infection. However, to address the limited evidence foundation for this critical topic the synthesis called for more refined model systems that could address questions related to aerosol size and respiratory tract deposition as they affect infection initiation and also determinants of susceptibility.

### Summary of the Science.

Discussions at the workshop were convergent on several critical findings relevant to control of airborne transmission: 1) SARS-CoV-2 is transmitted by aerosols; 2) effective measures are available to reduce transmission, including masks, social distancing, and ensuring sufficient building ventilation and using HEPA (high-efficiency particular air) filtration; and 3) layered interventions are needed to address the multiple pathways leading to infection by SARS-CoV-2 (Table 1). Interventions taken need to reflect the heterogeneity of environmental, population health, and social factors driving the inequitable burden of the pandemic, which has fallen heavily on the elderly, persons of color, and those with lower incomes.

**Table 1.** Layered interventions are needed to control transmission of SARS-CoV-2

• Masks limit bidirectional transfer of infectious particles, protecting the wearer and those surrounding the wearer, i.e., acting as source control.
• Ventilation and filtration can reduce aerosol concentrations indoors. There are far fewer cases of outdoor transmission than indoors, so gatherings should be outdoors if possible, as rapid dilution in outdoor air reduces the potential for exposure.
• Plexiglas barriers and face shields reduce droplet transmission but do not limit small aerosols that are transported in the air currents.
• Infectious particles on the floor, surfaces, clothing, and other objects can be resuspended in air if disturbed, with implications for cleaning protocols and handling of used PPE.
• Given the importance of spread of SARS-CoV-2 by infected hosts, the aerosol route is especially important in conversation at close distances and in crowded, poorly ventilated rooms.
• Evidence on aerosol generation, sizes, and concentrations indicates:
• universal masking and ventilation/filtration will reduce airborne concentrations of SARS-CoV-2.
• indoor activities should be limited, masks should be worn, and distances should be maintained at all times indoors when multiple people are present.

### Reach and Impact.

The two-day workshop in 2020 set records for the NASEM in its reach and breadth, with amplification by social media (namely Twitter) and nearly 15,000 live viewers and 10,900 Twitter engagements. Scientifically, the workshop format was successful in promoting needed multidisciplinary discussion, a goal of EHMI and the planning committee. In spring 2021, the WHO website and CDC guidance were updated to indicate aerosol inhalation as an important route of COVID-19 spread (10, 11) and a historical perspective on the issue was provided by Randall et al. (12). Three recent publications have reaffirmed the workshop’s concepts (13–15). The infrastructure and processes used for the workshop provide a rapid and inclusive response to emerging issues, strengthening the scientific basis for decisions and meeting the needs of policymakers and the public.
